# Identification of *Cis*-Acting Elements on Positive-Strand Subgenomic mRNA Required for the Synthesis of Negative-Strand Counterpart in Bovine Coronavirus

**DOI:** 10.3390/v6082938

**Published:** 2014-07-30

**Authors:** Po-Yuan Yeh, Hung-Yi Wu

**Affiliations:** Graduate Institute of Veterinary Pathobiology, College of Veterinary Medicine, National Chung-Hsing University, Taichung 40227, Taiwan; E-Mail: ybu999@gmail.com

**Keywords:** coronavirus, *cis*-acting element, subgenomic mRNA, negative-strand RNA synthesis

## Abstract

It has been demonstrated that, in addition to genomic RNA, sgmRNA is able to serve as a template for the synthesis of the negative-strand [(−)-strand] complement. However, the *cis*-acting elements on the positive-strand [(+)-strand] sgmRNA required for (−)-strand sgmRNA synthesis have not yet been systematically identified. In this study, we employed real-time quantitative reverse transcription polymerase chain reaction to analyze the *cis*-acting elements on bovine coronavirus (BCoV) sgmRNA 7 required for the synthesis of its (−)-strand counterpart by deletion mutagenesis. The major findings are as follows. (1) Deletion of the 5'-terminal leader sequence on sgmRNA 7 decreased the synthesis of the (−)-strand sgmRNA complement. (2) Deletions of the 3' untranslated region (UTR) bulged stem-loop showed no effect on (−)-strand sgmRNA synthesis; however, deletion of the 3' UTR pseudoknot decreased the yield of (−)-strand sgmRNA. (3) Nucleotides positioned from −15 to −34 of the sgmRNA 7 3'-terminal region are required for efficient (−)-strand sgmRNA synthesis. (4) Nucleotide species at the 3'-most position (−1) of sgmRNA 7 is correlated to the efficiency of (−)-strand sgmRNA synthesis. These results together suggest, in principle, that the 5'- and 3'-terminal sequences on sgmRNA 7 harbor *cis*-acting elements are critical for efficient (−)-strand sgmRNA synthesis in BCoV.

## 1. Introduction

During coronavirus replication, in addition to genomic RNA, a 3' coterminal nested set of subgenomic mRNAs (sgmRNAs), which are also 5' coterminal with the leader sequence of the genome, are synthesized via discontinuous transcription [1,2,3,4]. A mechanism by which sgmRNA acquires the leader sequence during negative-strand [(−)-strand)] synthesis using the positive-strand [(+)-strand] genomic RNA as a template has gained favor to explain this discontinuous step [5,6,7,8,9,10,11,12]. It has been demonstrated that both (−)- and (+)-strand sgmRNAs are present in coronavicus-infected cells [8]. Moreover, the 65–90-nt-long antileader sequence (depending on the species) on the (−)-strand sgmRNA has been proposed to be a promoter for the synthesis of its (+)-strand counterpart. It is therefore postulated that sgmRNA could be a replicon during coronavirus replication. However, no accumulation of the reporter-containing sgmRNA was found under the experimental condition in which the reporter-containing sgmRNA was transfected into helper-virus infected cells, suggesting that sgmRNA is not a replicon [13]. Regardless, using (+)-strand sgmRNA rather than (+)-strand genomic RNA as the template, it has been demonstrated that (−)-strand sgmRNA was synthesized and that smaller sgmRNA can also be produced if the sgmRNA template carried a transcription signal, suggesting that coronavirus is able to amplify sgmRNA via a mechanism other than replication [14]. Coronavirus sgmRNAs synthesized by this alternative mechanism may relieve pressure on the large genome, enhance disease development through the rapid amplification of virulence factors encoded by a large number of sgmRNAs, and contribute to the survival of the largest known viral RNA genome via sgmRNA-assisted recombination with the genome [15,16,17,18,19,20,21].

Due to the limited number of (−)-strand viral RNAs present in cells and insufficient methods for detecting and quantitating the synthesis of (−)-strand coronaviral RNA, little is known regarding the mechanism of how coronaviral (−)-strand RNA is generated [8]. For bovine coronavirus (BCoV), BCoV defective interfering (DI) RNA, a surrogate for the BCoV genome, has been used as a minireplicon for the study of *cis*-acting elements required for replication [interpreted as (+)-strand synthesis] [22,23,24,25,26,27,28,29,30,31]. Nonetheless, the identification of *cis*-acting elements required for (−)-strand BCoV DI RNA synthesis has been hampered due to inadequate methods for the detection of (−)-strand BCoV DI RNA. To overcome the technical problems, Wu and Brian have developed a head-to-tail ligation method and reverse transcription polymerase chain reaction (RT-PCR) to detect (−)-strand BCoV DI RNA and sgmRNA, demonstrating that both the BCoV DI RNA and sgmRNA (−) strands can be generated from their (+)-strand counterparts [14]. Accordingly, this may constitute a useful system for determining the *cis*-acting elements for the synthesis of BCoV (−)-strand DI RNA and sgmRNA [32].

In BCoV DI RNA, there are two putative stem-loop (SL) structures in the leader sequence which are part of the genome 5' UTR and required for the replication of BCoV DI RNA [13]. Downstream of the leader sequence in BCoV DI RNA is the 5'-proximal 421-nts region which includes 5' UTR and nsp1 coding sequence and is missing in sgmRNA 7, which corresponds to the sgmRNA encoding N protein. This region contains other higher-order RNA elements which have been determined to be the *cis*-acting elements important for the replication [22,27,28,33]. In the 3' UTR, two higher-order structures in the upstream region have been identified as *cis*-acting RNA elements essential for coronavirus replication, including a bulged stem-loop (BSL) [34,35] and a hairpin-type pseudoknot (PK) [31]. Further downstream of the 3' UTR is a hypervariable region (HVR) that has been demonstrated to be nonessential for RNA synthesis but does play a pivotal role in pathogenesis [36]. In coronaviruses, sgmRNA is structurally coterminal with the genome by the 5' leader sequence and the 3' UTR [1]. Because BCoV sgmRNA can serve as a template for the synthesis of its (−)-strand counterpart [14], we speculate that these *cis*-acting RNA structures on sgmRNA may also be involved in the process of (−)-strand RNA synthesis. In the present work, we identified the *cis*-acting elements on the (+)-strand sgmRNA that are required for (−)-strand sgmRNA synthesis using real-time quantitative reverse transcription polymerase chain reaction (RT-qPCR). Our findings indicate that the terminal sequences on the sgmRNA 5' and 3' UTRs are critical elements for efficient (−)-strand sgmRNA synthesis.

## 2. Materials and Methods

### 2.1. Viruses and Cells

A DI RNA-free stock of the Mebus strain of BCoV [GenBank accession no. U00735] at 3 × 10^7^ PFU/mL was used as a helper virus in the human rectal tumor (HRT)-18 cell line, as described previously [37,38].

### 2.2. Plasmid Constructs

Construction of pBM25A in which the 288-nt 3' UTR of BCoV-Mebus DI RNA in pDrep1 was replaced with the 301-nt 3' UTR and 25-nt poly(A) tail of MHV-A59 has been described [32]. To construct psBM25A, pNrep1, which encodes BCoV sgmRNA 7 [13], was digested with *NgoM*IV and *Xba*I, and the digested fragment containing the sgmRNA 7 5' leader sequence was cloned into *NgoM*IV and *Xba*I-linearized pBM25A, creating BCoV sgmRNA 7 with the 301-nt 3' UTR and 25-nt poly(A) tail of MHV-A59. A previously described overlap PCR mutagenesis procedure was used to construct sNL in which the leader sequence of sBM25A was deleted [5]: oligonucleotides pGEMNDEI(−) and sNL(+) and psBM25A DNA were used in the first PCR; oligonucleotides sNL(−) and RYN(+) and psBM25A DNA were used in the second PCR; and oligonucleotides pGEMNDEI(−) and RYN(+) and the products of the first two reactions were used in a third PCR to generate a 883-nt product that was cloned into the TOPO XL vector (Invitrogen). From this, a 462-nt fragment obtained by digestion with* NgoM*IV and *Xba*I was cloned into* NgoM*IV and *Xba*I-linearized psBM25A to produce psNL. Constructs of psΔSL1 and psΔSL2 in which stem-loop I and stem-loop II, respectively, was deleted were similarly generated except for the corresponding oligonucleotides used in the first and second reactions, as described in Table S1. Construct psΔB (deletion of 3' UTR bulged stem-loop) was produced following a previously described overlap PCR mutagenesis procedure but with oligonucleotides TGEV 7(−) and sΔB (+) and pBM25A DNA in the first PCR, oligonucleotides sΔB (−) and DI reverse(+) and pBM25A DNA in the second PCR, and oligonucleotides TGEV 7(−) and DI reverse(+) and the products of the first two reactions in a third PCR, generating a 1203-nt product that was cloned into the TOPO XL vector (Invitrogen). From this, a 756-nt fragment obtained by digestion with *Spe*I and *Mlu*I was cloned into *Spe*I and *Mlu*I-linearized psBM25A to produce the mutant psΔB. Constructs psΔP (deletion of 3' UTR pseudoknot), ps3'Δ55 (deletion of 3'-termianl 55 nts), psΔ3'15 (deletion of 3'-termianl 15 nts), psΔ3'55–40 (deletion of nts from −40 to −55), psΔ3'55–35 (deletion of nts from −35 to −55), psΔ3'55–30 (deletion of nts from −30 to −55), psΔ3'30–15 (deletion of nts from −15 to −30), psgmA’ (substitution of the 3'-most nt with adenine), psgmG’ (substitution of the 3'-most nt with guanine), and psgmU’ (substitution of the 3'-most nt with uracil) were similarly constructed except the corresponding oligonucleotides (Table S1) used in the first and second reactions.

### 2.3. RT-PCR for Detecting (−)-Strand RNA Products

*Mlu*I-linearized pBM25A and psBM25A were transcribed* in vitro* with RiboMAx T7 kit (Promega, Madison, WI, USA) in a total reaction volume of 50 μL. The reaction was performed at 37 °C for 90 min, treated with 5 μL DNase, and chromatographed through a Biospin 6 column (Bio-Rad, Hercules, CA, USA). For transfection, 3 µg of transcript, which was quantitated by denaturing gel electrophoresis, was transfected into HRT-18 cells with lipofectin (Invitrogen, Carlsbad, CA, USA) in 35-mm dishes at ~80% confluency (~8 × 10^5^ cells/dish), which were infected with BCoV at a multiplicity of infection of five PFU per cell 2 h prior to transfection [13] and virus within the transfected cells was referred to as virus passage 0 (VP0). To detect (−)-strand RNA, the total cellular RNA was extracted with TRIzol (Invitrogen) at 8 h posttransfection (hpt). Head-to-tail ligation of viral RNA and RT-PCR for detecting (−)-strand RNA have been described previously [14,39]. For this, 10 µg of extracted RNA was treated with 10 U (in 1 µL) of tobacco acid pyrophosphatase (Epicentre, Madison, WI, USA) in 25 µL of water and 3 µL of 10× buffer to remove the 5' capped end of the RNA. After phenol-chloroform extraction, the decapped RNA in 25 µL of water was heat-denatured at 95 °C for 5 min and then quick-cooled for 1 min. Then, 3 µL of 10× ligase buffer and 2U (in 2 µL) of T4 RNA ligase I (New England Biolabs, Ipswich, MA, USA) were added, and the mixture was incubated for 16 h at 16 °C. The ligated RNA was phenol-chloroform extracted and quantitated, and 1 µg of ligated RNA was used for the RT reaction to detect (−)-strand BM25A and sBM25A. Oligonucleotide MHV3'UTR3(−), which anneals at nts 89–112 from the poly(U) tail on the (−) strand of the MHV-A59 3' UTR, was used to synthesize cDNA with SuperScript III reverse transcriptase (Invitrogen). A 5-µL aliquot of this reaction was used in a 50-µL PCR with AccuPrime Tag DNA polymerase (Invitrogen) and oligonucleotides MHV3'UTR6(−), which anneals at nts 64–85 from the poly(U) tail on the (−) strand of the MHV-A59 3' UTR, and BCV23-40(+), which anneals at nts 23–40 on the (+)-strand leader of BCV. The mixture was heated to 94 °C for 2 min and then subjected to 50 cycles of 30 s at 94 °C, 30 s at 60 °C, and 30 s at 72 °C. The resulting RT-PCR products of ~150-base pair (bp) were directly sequenced. To detect the (−)-strand RNA without head-to-tail ligation step, 1 µg of RNA was used for RT-PCR reaction with oligonucleotides MHV3'UTR-DR(−) (for RT), which binds to nts 140–160 from the poly(U) tail in the (−) strand of the MHV-A59 3' UTR and MHV3UTR-DR(+), which binds to nts 56–73 from the poly(U) tail in the (−) strand of the MHV-A59 3' UTR. The mixture was heated to 94 °C for 2 min and then subjected to 25 cycles of 30 s at 94 °C, 30 s at 60 °C, and 30 s at 72 °C.

### 2.4. Quantitation of (−)-Strand sgmRNA Synthesis by RT-qPCR

To assess the efficiency of (−)-strand sgmRNA synthesis from wt sBM25A and the mutants except sNL, sΔSL1 and sΔSL2, 1 µg of decapped and ligated RNA collected from BCoV-infected sgmRNA-transfected HRT-18 cells at 8 hpt was used in an RT reaction with oligonucleotide MHV3'UTR3(−) and SuperScript III reverse transcriptase (Invitrogen). TaqMan probe-5 (Table S1) used for RT-qPCR were designed by the Primer Express computer program (Applied Biosystems, Foster City, CA, USA). The real-time PCR amplification was performed in a LightCycler^®^ 480 instrument (Roche Applied Science, Indianapolis, IN, USA) using MHV3'UTR6(−) and BCV23-40(+) primers, and TaqMan^®^Universal PCR Master Mix (Applied Biosystems) according to the manufacturer’s recommendations. To assess the efficiency of (−)-strand sgmRNA synthesis from wt sBM25A and the mutants sNL, sΔSL1 and sΔSL2, 1 µg of RNA with decapping and head-to-tail ligation was used for RT reaction with oligonucleotide MHV3'UTR-DR(−) to synthesize cDNA with SuperScript III reverse transcriptase (Invitrogen). TaqMan^®^Universal PCR Master Mix (Applied Biosystems) with TaqMan probe-3 and primers MHV3UTR-DR(-) and BCVN(+) was used for RT-qPCR. To assess the synthesis of (−)-strand RNA from RNA samples without decapping and head-to-tail ligation, 1 µg of RNA was used for RT reaction with oligonucleotide MHV3'UTR-DR(−) to synthesize cDNA with SuperScript III reverse transcriptase (Invitrogen). TaqMan^®^Universal PCR Master Mix (Applied Biosystems) with TaqMan probe-3 and primers MHV3UTR-DR(−) and MHV3UTR-DR(+) (Table S1), which binds to nts 56–73 from the poly(U) tail in the (−) strand of the MHV-A59 3' UTR, was used for RT-qPCR. In either of the experiments, dilutions of plasmids containing the same gene as the detected (−)-strand RNA were always run in parallel with the quantitated cDNA for use as standard curves (the dilutions ranged from 10^8^–10 copies of each plasmid). The efficiency of (−)-strand RNA synthesis was normalized with the levels of internal controls, including 18S rRNA (with primers 18SrRNA(−) and 18SrRNA(+)), (+)-strand reporter-containing sgmRNA (with primers MHV3UTR-DR(−) and MHV3UTR-DR(+)) and helper virus M sgmRNA (with primers leader20(−) and M(+)) extracted at 8 hpt (VP0). The reactions were performed as an initial pre-incubation at 95 °C for 5 min, followed by 35 amplification cycles of 95 °C for 15 s and 60 °C for 60 s.

### 2.5. Northern Blot Assay for DI RNA Replication

A Northern blot assay was performed as described previously [11,13]. Briefly, HRT-18 cells in 35-mm dishes at ~80% confluency (~8 × 10^5^ cells/dish) were infected with BCoV at a multiplicity of infection of five PFU per cell. Two hours post-infection (hpi), 3 µg of transcript was transfected into the HRT-18 cells. To detect the replication of BCoV DI RNA and sgmRNAs, the supernatant was collected at 48 hpt and then used to infect fresh HRT-18 cells (virus passage 1, VP1). Ten µg of TRIzol-extracted total cellular RNA at 48 hpi of VP1 was used for formaldehyde-agarose gel electrophoresis. To detect 18S rRNA, reporter-containing sgmRNA, and M sgmRNA, 10 µg of TRIzol-extracted total cellular RNA at 8 hpt (VP0) was used for formaldehyde-agarose gel electrophoresis. RNA was then transferred from the gel to Nytran membranes by vacuum blotting. The blots were probed with digoxigenin (DIG)-ddUTP labeled (DIG Oligonucleotide 3'-End Labeling kit; Roche Molecular Biochemicals) oligonucleotides: TGEV 8(+) (for reporter-containing BM25A, sBM25A, and sgmRNA), BCVN(+) (for M sgmRNA), and 18SrRNA(+) (for 18S rRNA). The detected RNA was visualized according to the procedure of the manufacturer.

### 2.6. Statistical Analyses

Statistical calculations were performed using GraphPad Prism v2.01 [40]. *p* values were determined with paired *t* test for RT-qPCR data.

## 3. Results

### 3.1. The (−)-Strand Synthesis from sgmRNA 7 Is ~2-Fold less than that from BCoV DI RNA, a Surrogate for BCoV Genome

**Figure 1:**
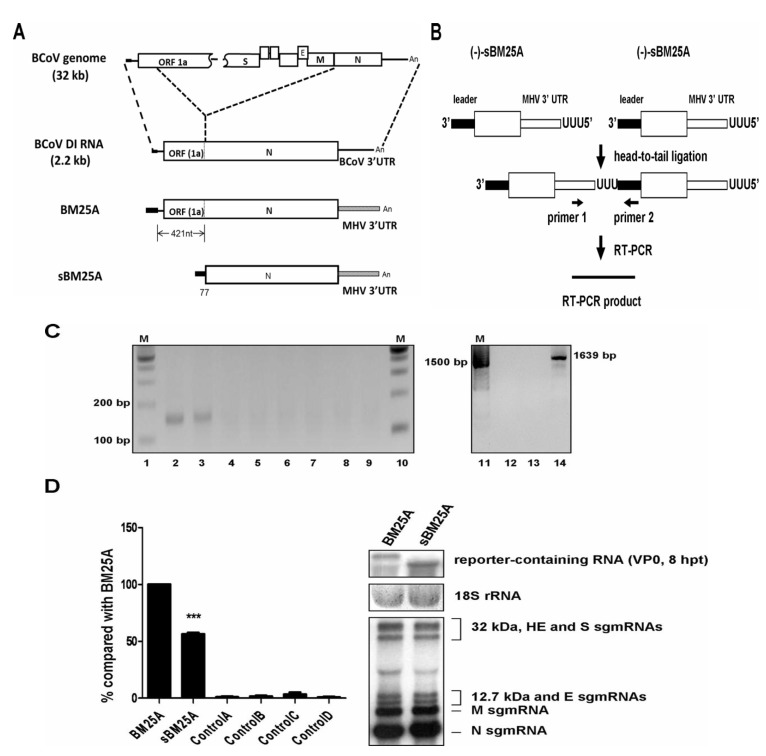
Comparison of the efficiency of the (−)-strand RNA synthesis between sgmRNA 7 and BCoV DI RNA with the strategy of head-to-tail ligation. (**A**) Upper panel: schematic diagram depicting the BCoV genome and BCoV DI RNA. Lower panel: constructs BM25A and sBM25Awith the 3' UTR of both constructs replaced with the MHV 3' UTR. (**B**) Strategy for detection of (−)-strand sgmRNA with head-to-tail ligation. (**C**) RT-PCR product synthesized with the strategy described in Figure 1B. Lane 2, BM25A-transfected BCoV-infected cells; lane 3, sBM25A-transfected BCoV-infected cells; lane 4, mock-infected cells; lane 5, BCoV-infected and mock-transfected cells; lane 6, BM25A-transfected mock-infected cells; lane 7, sBM25A-transfected mock-infected cells; lane 8, a mixture of BCoV-infected cellular RNA extracted at 8 hpt and 200 ng of BM25A transcript; lane 9, a mixture of BCoV-infected cellular RNA extracted at 8 hpt and 200 ng of sBM25A transcript. RT-PCR was used to detect the potential recombination between the BCoV genome and BM25A (lane 12) or sBM25A (lane 13). A recombinant DNA of 1639 bp was produced to serve as a size marker (lane 14). M (lanes 1, 10 and 11), ds DNA size markers in nt pairs. (**D**) Left panel: the relative efficiency of (−)-strand RNA synthesis from constructs BM25A and sBM25A, as measured by RT-qPCR. Control A: total cellular RNA from mock-infected cells. Control B: total cellular RNA from BCoV-infected cells. Control C: total cellular RNA from sBM25A-transfected mock-infected cells. Control D: a mixture of BCoV-infected cellular RNA extracted at 8 hpt and 200 ng of BM25A transcript. Right panel: measurements of reporter-containing RNA (BM25A and sBM25A), 18S rRNA and BCoV sgmRNAs at 8 hpt (VP0) by Northern blot assay. The values (D) represent the mean ± SD of three individual experiments. *** *p* < 0.001.

To study coronavirus replication, an ~ 2.2 kb BCoV DI RNA has been employed as a surrogate for the ~30 kb BCoV genome (Figure 1A, upper panel) [10,11,13,22,23,27,28,29,31,33]. In DI RNA-transfected BCoV-infected cells at 24 hpi of VP1, however, there are only an estimated ~5 molecules of BCoV DI RNA (−) strand per cell [11,41]. Therefore, to overcome (1) the detection problem due to the low copy number of (−)-strand BCoV DI RNA in infected cells and (2) the false positive results of (−)-strand DI RNA detection caused by copy-back (−)-strand DI RNA transcripts generated by T7 RNA polymerase using plasmid DNA as a template [42], a head-to-tail ligation method and reverse transcription polymerase chain reaction (RT-PCR) (Figure 1B) have been developed to detect and quantitate the synthesis of (−)-strand BCoV DI RNA [14,32,39]. With this method, it has been demonstrated that both BCoV DI RNA and BCoV sgmRNA 7 can function as the template for the synthesis of their (−)-strand copies [14], even though sgmRNA 7 lacks the 5'-proximal 421 nts in comparison to the genome surrogate BCoV DI RNA. The relative efficiency of (−)-strand synthesis between the two RNA species, however, has not been previously described. Thus, to assess the efficiency of (−)-strand RNA synthesis between the two species and then to determine the requirement of the 5'-proximal 421 nts for the synthesis of (−)-strand viral RNA, constructs BM25A, which represents BCoV DI RNA, and sBM25A, which represents sgmRNA 7 (Figure 1A, lower panel), were generated. The BCoV 3’ UTR from either of the constructs was replaced with the MHV 3´ UTR to distinguish them from the helper virus during detection. The transcripts of BM25A and sBM25A were separately transfected into BCoV-infected HRT-18 cells and cellular RNA was extracted at 8 hpt. The extracted RNA was decapped, head-to-tail ligated, and subjected to RT-PCR with primers MHV3'UTR6(−) and BCV23-40(+) (Figure 1B). An ~150-bp RT-PCR product was detected from the BCoV-infected cells transfected with BM25A or sBM25A (Figure 1C, lanes 2–3) but not from the mock-infected cells, BCoV-infected cells, transfected mock-infected cells, or in control reactions with mixed components of infected cell RNA and input BM25A or sBM25A (Figure 1C, lanes 4–9). Taken together, these results demonstrated that the RT-PCR products are specifically from putative viral polymerase-generated (−)-strand RNA. Furthermore, it has been shown that the input DI RNA and coronavirus genome may undergo recombination under certain selection pressure [15,16,43]. Therefore, the detected reporter-containing (−)-strand DI RNA or sgmRNA may be derived from a recombinant molecule containing the coronavirus genome and reporter-containing DI RNA or sgmRNA genes. To ensure that the detected (−)-strand RNA was specifically from reporter-containing BM25A or sBM25A rather than from a recombinant molecule, RT-PCR using primers that anneal to the reporter sequence in BM25A or sBM25A (for RT) and the M protein gene in the BCoV genome was performed to test for a potential recombinant molecule generated during infection [14]. No RT-PCR product was observed (Figure 1C, lanes 12–13), thus excluding the possibility of the detected (−)-strand RNA species being synthesized from a recombinant. Note that a recombinant DNA of 1639 bp (Figure 1C, lane 14) was produced to serve as a size marker [14,32,44] for a potential RT-PCR product in Figure 1C, lanes 12–13. To measure the efficiency of (−)-strand RNA synthesis from BM25A and sBM25A, RT-qPCR was performed with 18S rRNA, input (+)-strand BM25A, or sBM25A and M sgmRNA of helper virus BCoV as internal controls (Figure 1D, left panel). Besides, to ensure that the (−)-strand RNA was synthesized under the similar experimental conditions, the level of reporter-containing (+)-strand BM25A or sBM25A, 18S rRNA, and sgmRNAs of helper virus BCoV at 8 hpt (VP0) was also determined with Northern blot assay (Figure 1D, right panel). As shown in Figure 1D (left panel), the efficiency of (−)-strand RNA synthesis was ~2-fold less from sBM25A than from BM25A (56% *vs.* 100%) under the similar experimental conditions.

Considering that the inefficient head-to-tail ligation may decrease the numbers of (−)-strand RNA species to be quantitated and thus may affect the subsequent interpretation of the results, RT-qPCR was also performed using RNA without ligation reaction with primers annealing to the hypervariable region of MHV 3' UTR. Although the false positive results of (−)-strand RNA detection caused by copy-back (−)-transcripts generated by T7 RNA polymerase may occur, the levels of copy-back molecules determined by RT-qPCR from uninfected cells transfected with transcripts can be used as the background levels of (−)-strand RNA for BM25A and sBM25A. The relative quantitation of (−)-strand RNA synthesis from BM25A and sBM25A compared to the background levels would suggest that the increase of the (−)-strand RNA synthesis is due to the activity of viral RNA-dependent RNA polymerase. As shown in Figure S1A, 1 µg of RNA without decapping and head-to-tail ligation was used for RT-PCR with primers MHV3UTR-DR(−) and MHV3UTR-DR(+), both of which bind to the hypervariable region of MHV 3' UTR. A ~100-bp RT-PCR product was detected from the BCoV-infected cells transfected with BM25A or sBM25A (lanes 2–3) but not from the mock-infected cells (lane 4) and BCoV-infected cells (lane 5). The ~100-bp RT-PCR product was also detected from uninfected cells transfected with BM25A or sBM25A (lanes 6–7), suggesting the RT-PCR product was caused by copy-back (−)-transcripts generated by T7 RNA polymerase. To measure the relative efficiency of (−)-strand RNA synthesis from BM25A and sBM25A, RT-qPCR was performed without the ligation of RNA. As shown in Figure S1B, the efficiency of the (−)-strand RNA synthesis from sBM25A was also ~2-fold less than that from BM25A (~60% of BM25A), confirming the previous results in which RNA samples were head-to-tail ligated prior to RT-qPCR. Taken together, these results suggest that the (−)-strand synthesis from sgmRNA 7 (sBM25A) is ~2-fold less than that from BCoV DI RNA (BM25A), a surrogate for BCoV genome, and thus the 5'-proximal 421-nt sequence is required for optimal (−)-strand RNA synthesis in BCoV.

### 3.2. Screening for cis-Acting Elements in the 5' and 3' UTRs Required for (−)-Strand sgmRNA 7 Synthesis

**Figure 2:**
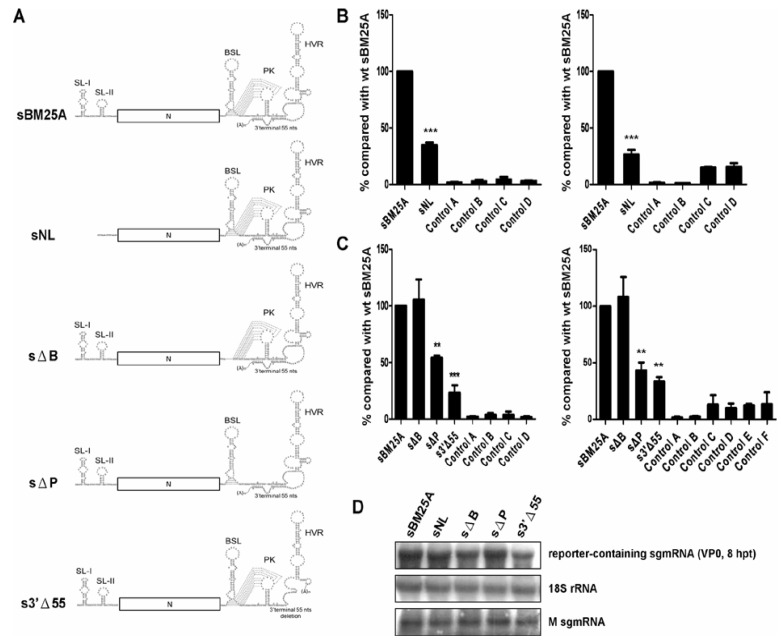
Identification of the *cis*-acting elements within the 5' and 3' UTRs of sgmRNA that are required for (−)-strand sgmRNA synthesis. (**A**) Illustration of the constructs with deletions in the 5' UTR (construct sNL) and 3' UTR (constructs sΔB, sΔP, and s3'Δ55). SL: stem-loop; BSL: bulged stem-loop; PK: pseudoknot; HVR: hypervariable region. (**B**) The relative efficiency of (−)-strand sgmRNA synthesis, as measured by RT-qPCR with (left panel) or without (right panel) head-to-tail ligation. Left panel: Control A: total cellular RNA from mock-infected cells. Control B: total cellular RNA from BCoV-infected cells. Control C: total cellular RNA from sBM25A-transfected mock-infected cells. Control D: a mixture of BCoV-infected cellular RNA extracted at 8 hpt and 200 ng of sBM25A transcript. Right panel: Control A: total cellular RNA from mock-infected cells. Control B: total cellular RNA from BCoV-infected cells. Control C: total cellular RNA from sBM25A-transfected mock-infected cells. Control D: total cellular RNA from sNL -transfected mock-infected cells. (**C**) The relative efficiency of (−)-strand sgmRNA synthesis, as measured by RT-qPCR with (left panel) or without (right panel) head-to-tail ligation. Left panel: Control A: total cellular RNA from mock-infected cells. Control B: total cellular RNA from BCoV-infected cells. Control C: total cellular RNA from sBM25A-transfected mock-infected cells. Control D: a mixture of BCoV-infected cellular RNA extracted at 8 hpt and 200 ng of sBM25A transcript. Right panel: Control A: total cellular RNA from mock-infected cells. Control B: total cellular RNA from BCoV-infected cells. Control C: total cellular RNA from sBM25A-transfected mock-infected cells. Control D: total cellular RNA from sΔB-transfected mock-infected cells. Control E: total cellular RNA from sΔP-transfected mock-infected cells. Control F: total cellular RNA from s3'Δ55-transfected mock-infected cells. (**D**) Measurements of reporter-containing sgmRNA, 18S rRNA and M sgmRNA (from helper virus) at 8 hpt of VP0 by Northern blot assay. M sgmRNA rather than N sgmRNA was chosen to represent helper virus here and in the following figures because the reporter-containing (+)-strand sgmRNA and BCoV N sgmRNA (sgmRNA 7) migrated to the same position. The values (B) and (C) represent the mean ± SD of three individual experiments. ** *p* < 0.01, *** *p* < 0.001.

Structurally, sgmRNAs and genomic RNA in BCoV share common features at the termini, including a 5' leader sequence and 3' UTR. In addition to the leader sequence [13] within the 5' UTR, the bulged stem-loop [34,35] and the hairpin-type pseudoknot [31] within the 3' UTR have been demonstrated to be *cis*-acting elements required for coronavirus replication. Furthermore, the 3'-terminal 55-nt sequence has been shown to be critical for synthesis of (−)-strand MHV DI RNA [26]. However, these structures on (+)-strand sgmRNA have not been previously shown to be required for (−)-strand sgmRNA synthesis in BCoV. Thus, a series of sgmRNA mutants were constructed to examine the requirement of these RNA elements on (+)-strand sgmRNA for (−)-strand sgmRNA synthesis, including deletions of the leader sequence (sNL), bulged stem-loop (sΔB), pseudoknot (sΔP), and 3'-terminal 55 nts (s3'Δ55) (Figure 2A). As the hypervariable region is not conserved among coronaviruses and has been shown to be nonessential for coronavirus replication [36], this region was not tested in this study. Transcripts of each mutant were separately transfected into BCoV-infected HRT-18 cells, and cellular RNA was extracted at 8 hpt and subjected to RT-qPCR. To determine the efficiency of (−)-strand synthesis from leader-deleted construct sNL (Figure 2A), 1 µg of RNA with head-to-tail ligation was used for RT-qPCR (Figure 2B, left panel) with primers MHV3UTR-DR(−) and BCVN(+) because the primer BCV23-40(+) used in the above-described studies (Figure 1D) cannot anneal to the (−) strand of sNL. Besides, 1 µg of RNA without head-to-tail ligation was also used for RT-qPCR (Figure 2B, right panel) with primers MHV3UTR-DR(−) and MHV3UTR-DR(+). As shown in Figure 2B, the synthesis of (−)-strand sgmRNA from the leader sequence-deleted construct sNL was inhibited (~30% of wt sBM25A). To measure the efficiency of the deletion constructs sΔB, sΔP and s3'Δ55, RT-qPCR with (Figure 2C, left panel) or without (Figure 2C, right panel) previous head-to-tail ligation, primers MHV3'UTR6(−) and BCV23-40(+), or primers MHV3UTR-DR(−) and MHV3UTR-DR(+), were used respectively. As shown in Figure 2C, synthesis of the (−)-strand sgmRNA from the pseudoknot-deleted construct sΔP was inhibited (~50% of wt sBM25A). Surprisingly, (−)-strand sgmRNA synthesis from the sgmRNA construct with deletions of bulged stem-loop (sΔB) was not blocked. In contrast, the terminal 55-nt deletion in sgmRNA (s3'Δ55) significantly impaired the synthesis of its (−)-strand counterpart (~20% of wt sBM25A). To further determine whether the variation in the synthesis of (−)-strands among these constructs were due to changes in the number of (+)-strand templates resulting from replication, replication [interpreted as (+)-strand RNA synthesis] of wt sBM25A and sgmRNA mutants was analyzed by Northern blot assay from total cellular RNA extracted at 48 hpi of VP1. As shown in Figure S2, no accumulation of the (+)-strand reporter-containing sgmRNA was found, suggesting the efficiency of (−)-strand synthesis among these sgmRNA constructs was not affected by the number of (+)-strand templates due to replication. Since (1) the synthesis of the (−)-strand counterpart occurred under the similar experimental conditions as determined by the level of reporter-containing (+)-strand sgmRNA constructs, 18S rRNA, and M sgmRNA of helper virus BCoV at 8 hpt (VP0) with Northern blot assay (Figure 2D) and (2) these sgmRNA constructs were dissemination-defective (Figure S2), these results suggest that the 5' leader sequence along with the 3'-terminal 55 nts and higher-order structure pseudoknot in sgmRNA are critical *cis*-acting elements required for (−)-strand sgmRNA synthesis.

### 3.3. Deletion of Individual Stem-Loops within the Leader Sequence of sgmRNA 7 only Shows Minor Effects on (−)-Strand sgmRNA Synthesis

**Figure 3:**
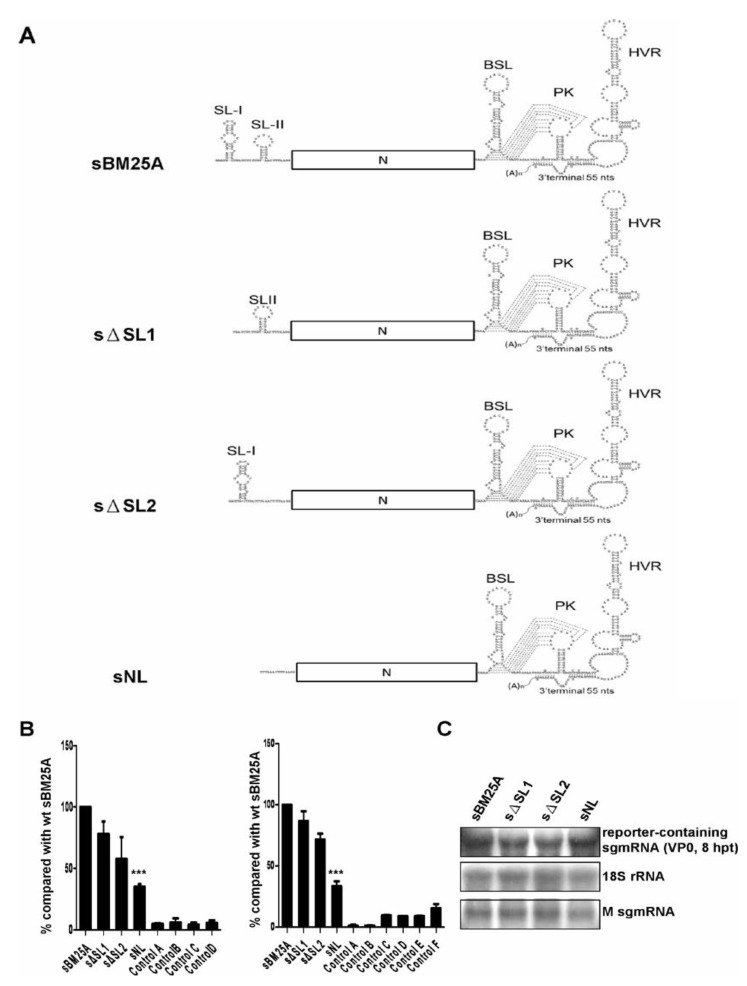
Identification of *cis*-acting elements within the leader sequence that are essential for (−)-strand sgmRNA synthesis. (**A**) Constructs with deletions in the leader sequence of wt sBM25A. sΔSL1: deletion of stem-loop I; sΔSL2: deletion of stem-loop II; sNL: deletion of the leader sequence. SL: stem-loop; BSL: bulged stem-loop; PK: pseudoknot; HVR: hypervariable region. (**B**) The relative efficiency of (−)-strand RNA synthesis between the constructs, as measured by RT-qPCR with (left panel) or without (right panel) head-to-tail ligation. Left panel: Control A: total cellular RNA from mock-infected cells. Control B: total cellular RNA from BCoV-infected cells. Control C: total cellular RNA from sBM25A-transfected mock-infected cells. Control D: a mixture of BCoV-infected cellular RNA extracted at 8 hpt and 200 ng of sBM25A transcript. Right panel: Control A: total cellular RNA from mock-infected cells. Control B: total cellular RNA from BCoV-infected cells. Control C: total cellular RNA from sBM25A-transfected mock-infected cells. Control D: total cellular RNA from sΔSL1-transfected mock-infected cells. Control E: total cellular RNA from sΔSL2-transfected mock-infected cells. Control F: total cellular RNA from sNL-transfected mock-infected cells. (**C**) Measurements of reporter-containing sgmRNA, 18S rRNA and M sgmRNA (from helper virus) at 8 hpt of VP0 by Northern blot analysis. The values (B) represent the mean ± SD of three individual experiments. *** *p*<0.001.

Although it has been demonstrated for MHV DI RNA that the 5' UTR, including the leader sequence, does not affect the synthesis of (−)-strand sgmRNA [26], we found here in this study that the leader sequence was required for the efficient synthesis of BCoV (−)-strand sgmRNA 7 (Figure 2B). The leader sequence in sgmRNA 7 of BCoV forms two stem-loops [13]; however, their role in the (−)-strand sgmRNA synthesis remains to be determined. To further dissect the *cis*-acting elements within the leader sequence required for (−)-strand sgmRNA synthesis, we generated constructs sΔSL1 and sΔSL2 in which stem-loop I and stem-loop II, respectively, was deleted (Figure 3A) and performed RT-qPCR with the head-to-tail RNA ligation step using primers MHV3UTR-DR(−) and BCVN(+) because the primer BCV23-40(+) used for RT-qPCR in the above-described studies cannot anneal to the (−) strand of sNL and sΔSL1. RT-qPCR was also used without the head-to-tail RNA ligation step using primers MHV3UTR-DR(−) and MHV3UTR-DR(+) to measure the efficiency of (−)-strand sgmRNA synthesis. As shown in Figure 3B, with (left panel) or without (right panel) the step of head-to-tail RNA ligation, the efficiency of (−)-strand sgmRNA synthesis from either of sΔSL1 and sΔSL2 was only slightly decreased in comparison to that from wt sBM25A under the similar experimental conditions (Figure 3C), whereas (−)-strand sgmRNA synthesis was still inhibited from sNL, the construct in which the leader sequence was deleted. These results suggest stem-loop I or stem-loop II alone still supports efficient (−)-strand sgmRNA synthesis; however, (−)-strand sgmRNA synthesis is significantly inhibited when both structures are missing.

### 3.4. The 3'-Terminal nts Positioned from −15 to −34 is Required for Efficient (−)-Strand sgmRNA Synthesis

**Figure 4:**
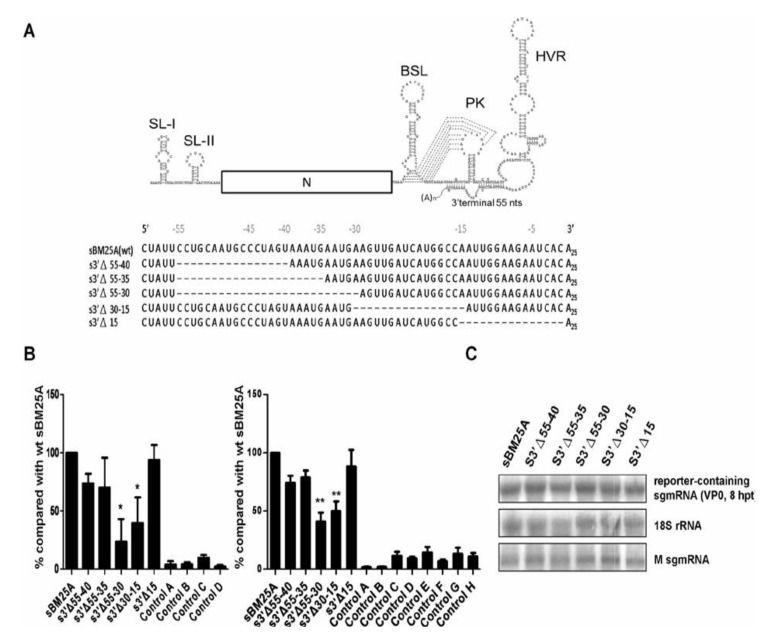
Mapping the *cis*-acting elements within 3'-terminal 55 nts that are required for (−)-strand sgmRNA synthesis. (**A**) Upper panel: schematic diagram depicting the sgmRNA. The 3'-terminal 55 nts are indicated. Lower panel: constructs of sgmRNA with deletions within the 3'-terminal 55 nts. SL: stem-loop; BSL: bulged stem-loop; PK: pseudoknot; HVR: hypervariable region. (**B**) The relative efficiency of (−)-strand RNA synthesis between the constructs, as measured by RT-qPCR with (left panel) or without (right panel) head-to-tail ligation. Left panel: Control A: total cellular RNA from mock-infected cells. Control B: total cellular RNA from BCoV-infected cells. Control C: total cellular RNA from sBM25A-transfected mock-infected cells. Control D: a mixture of BCoV-infected cellular RNA extracted at 8 hpt and 200 ng of sBM25A transcript. Right panel: Control A: total cellular RNA from mock-infected cells. Control B: total cellular RNA from BCoV-infected cells. Controls C–H: total cellular RNA from mock-infected cells transfected with sBM25A, s3'Δ55–40, s3'Δ55–35, s3'Δ55–30, s3'Δ30–15 and s3'Δ15, respectively. (**C**) Measurements of reporter-containing sgmRNA, 18S rRNA and M sgmRNA (from helper virus) at 8 hpt of VP0 by Northern analysis. The values (B) represent the mean ± SD of three individual experiments. * *p* < 0.05, ** *p* < 0.01.

Using MHV DI RNA, it has been concluded that the 3'-terminal 55 nts are essential for (−)-strand DI RNA synthesis [26]. We also demonstrated in previous [32] and present (Figure 2C) studies that the 3'-terminal 55 nts in BCoV DI RNA and sgmRNA 7, respectively, are required for (−)-strand RNA synthesis. To map the specific sequence within the 3'-terminal 55-nt region of sgmRNAs that is critical for synthesis of (−)-strand sgmRNA, a series of deletion mutants were constructed (Figure 4A) and tested using RT-qPCR. Constructs were tested with (Figure 4B, left panel) or without ( 4C, right panel) prior head-to-tail RNA ligation with primers MHV3UTR6(−) and BCV23-40(+), or primers MHV3UTR-DR(−) and MHV3UTR-DR(+), respectively. As shown in Figure 4B, under the similar experimental conditions (Figure 4C), deletion mutant s3'Δ15 in which the 3'-most 15 nts were deleted still maintained the ability to synthesize its (−)-strand counterpart, as did the mutants s3'Δ55–35 and s3'Δ55–40; however, the (−)-strand sgmRNA synthesis in mutants s3'Δ55–30 and s3'Δ30–15 was significantly inhibited, suggesting that the sequence positioned from −15 to −34 within the 3'-terminal 55 nts is a *cis*-acting element required for the efficient synthesis of (−)-strand sgmRNA.

### 3.5. The 3'-Most Nucleotide Species of sgmRNA 7 Affects the Efficiency of (−)-Strand sgmRNA Synthesis

**Figure 5:**
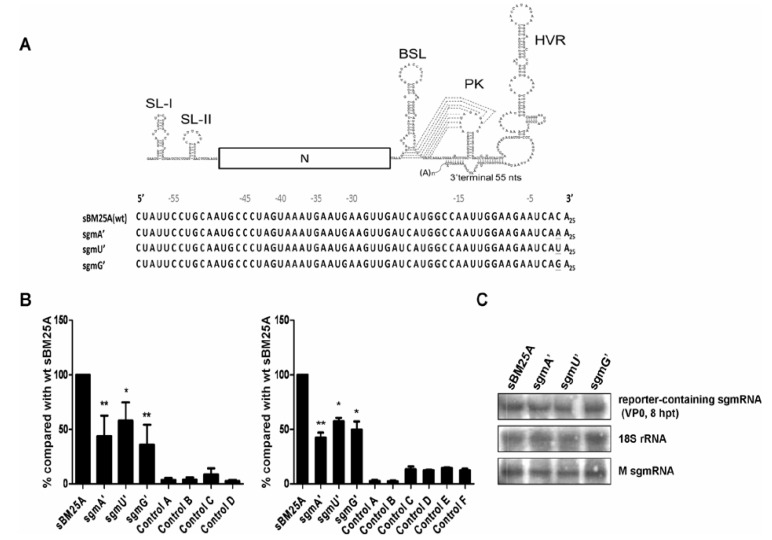
The effect of the 3'-most nt species on the synthesis of (−)-strand sgmRNA. (**A**) Upper panel: schematic diagram depicting the sgmRNA. Lower panel: constructs with nucleotide substitution at the 3'-most nt of sgmRNA. (**B**) The relative efficiency of (−)-strand RNA synthesis between the constructs, as measured by RT-qPCR with (left panel) or without (right panel) head-to-tail ligation. Left panel: Control A: total cellular RNA from mock-infected cells. Control B: total cellular RNA from BCoV-infected cells. Control C: total cellular RNA from sBM25A-transfected mock-infected cells. Control D: a mixture of BCoV-infected cellular RNA extracted at 8 hpt and sBM25A transcript. Right panel: Control A: total cellular RNA from mock-infected cells. Control B: total cellular RNA from BCoV-infected cells. Control C: total cellular RNA from sBM25A-transfected mock-infected cells. Control D: total cellular RNA from sgmA’-transfected mock-infected cells. Control E: total cellular RNA from sgmU’-transfected mock-infected cells. Control F: total cellular RNA from sgmG’-transfected mock-infected cells. (**C**) Measurements of reporter-containing sgmRNA, 18S rRNA and M sgmRNA (from helper virus) at 8 hpt of VP0 by Northern analysis. Values (B) represent the mean ± SD of three individual experiment s. * *p* < 0.05, ** *p* < 0.01.

The last nucleotide of the 3'UTR in all coronavirus genomes and subgenomes sequenced to date is cytosine. Therefore, this conserved nucleotide may play an important role during coronavirus infection. Since this nucleotide positions at the 3'-terminus of genome and subgenome, we speculated that it may be involved in (−)-strand RNA synthesis. To test whether this nucleotide correlates with the efficiency of (−)-strand sgmRNA synthesis, the cytosine was substituted with adenine, uracil, and guanine to create constructs sgmA’, sgmU’, and sgmG’, respectively (Figure 5A). As shown in Figure 5B, using RT-qPCR with (left panel) or without (right panel) the head-to-tail RNA ligation with primers MHV3UTR6(−) and BCV23-40(+) or primers MHV3UTR-DR(−) and MHV3UTR-DR(+), respectively, the efficiency of (−)-strand synthesis from construct sgmA’, sgmU’ and sgmG under the similar experimental conditions (Figure 5C) was decreased in comparison with that from wt sBM25A. These results suggest that the nucleotide species at the 3'-most position is correlated to the efficiency of (−)-strand sgmRNA synthesis.

## 4. Discussion

The previously published study has shown that (+)-strand sgmRNA can function as a template for the synthesis of (−)-strand counterpart [14]; however, the efficiency of (−)-strand synthesis using (+)-strand sgmRNA 7 as a template remains unknown. In the present study, we have demonstrated that the efficiency of (−)-strand sgmRNA synthesis from sgmRNA 7 is ~50% less than that from BCoV DI RNA (Figure 1D), a surrogate for the ~30 kb BCoV genome. The result that (+)-strand sgmRNAs still maintain the ability to synthesize their (−)-strand copies is not surprising since sgmRNA equips the complete secondary structures in 3' UTR required for the initiation of (−)-strand synthesis based on the secondary structure model proposed by Züst* et al.* [45] and thus the 5'-end genome-specific 412 nts which differentiate DI RNA from sgmRNA (Figure 1A) might function as an enhancer in the initiation of (−)-strand synthesis. Although the efficiency of (−)-strand synthesis from sgmRNA is impaired, the overwhelming abundance of total sgmRNAs in BCoV-infected cells (~6000 and ~20 molecules for sgmRNA and genome per cell, respectively) [41] may suggest that there are a number of (−)-strand sgmRNAs synthesized with (+)-strand sgmRNAs as templates, reinforcing the biological significance of (−)-strand sgmRNA synthesis using (+)-strand sgmRNAs as templates. Furthermore, the finding that (+)-strand sgmRNA with a transcription signal can be employed as a template to synthesize smaller sgmRNAs has also emphasized the additional function of coronavirus sgmRNAs [14]. As many of the *cis*-acting elements in the coronavirus genome required for replication have been identified in the past two decades [22,23,24,27,28,31,33,34,35,46,47,48,49,50,51], it is also important to examine the RNA elements on the (+)-strand sgmRNA that are required for the synthesis of its (−)-strand counterpart. In the present study, we identified the *cis*-acting elements on the (+)-strand sgmRNA that are essential for efficient (−)-strand sgmRNA synthesis, and these findings have expanded our knowledge of coronavirus replication.

It appears that the main function of the BCoV DI RNA 5'-proximal 421 nts, which is missing in sgmRNA, is for (+)-strand DI RNA synthesis because (1) both DI RNA and sgmRNA are able to generate their (−)-strand counterparts and (2) only (+)-strand DI RNA can be synthesized from the template of DI RNA (−) strand. In addition, since the efficiency of (−)-strand sgmRNA synthesis from sgmRNA is ~50% less than that from BCoV DI RNA (Figure 1D) and is ~3-fold higher than that from construct sNL in which both the leader sequence and 5'-proximal 421 nts are missing (Figure 2B), the efficiency of (−)-strand synthesis from BCoV DI RNA therefore is estimated to be ~6-fold higher than that from construct sNL. These results indicate that the 5'-terminal 498 nts of the coronavirus genome, including the leader sequence, are required for efficient (−)-strand RNA synthesis. This conclusion is different from a previously published work which suggested that the 5'-terminal sequence is not required for efficient (−)-strand RNA synthesis in MHV DI RNA using a ribonuclease protection assay [26]. The reasons for the different findings may be largely associated with the structural components and the replication mechanism between the two DI RNAs and are explained as follows. (1) MHV DI RNA consists of both MHV 5' and 3' UTRs and a foreign CAT gene, but the components of BCoV DI RNA are all from the BCoV genome, including the BCoV 5' UTR, 3' UTR, part of the nsp 1 gene, and intact N gene. (2) It has been demonstrated that the replication of BCoV DI RNA is translation-dependent [52], indicating the BCoV DI RNA fusion protein and potential interaction between the fusion protein and structures on the DI RNA are important for replication, including (−)-strand DI RNA synthesis; however, *cis*-acting proteins are not required for the replication of MHV DI RNA [53]. It has been demonstrated, using a complete reverse genetic system, that SL I and SL II in the 5' UTR of MHV-A59 genome are required for (−)-strand sgmRNA synthesis [47,48], whereas using BCoV sgmRNA 7 and RT-qPCR we have demonstrated in the present study that the deletion of either of the SLs in the context of sgmRNA 7 still supports (−)-strand sgmRNA synthesis (Figure 3B). We speculate that SL I or SL II alone may still maintain part of secondary structure required for (−)-strand sgmRNA synthesis and that is why the efficiency of (−)-strand synthesis from either of the deletion constructs was only slightly impaired. However, once the entire secondary structure required for (−)-strand sgmRNA synthesis was missing, the synthesis of (−)-strand sgmRNA was significantly blocked as evidenced by the results from sNL (Figure 3B). Alternatively, regarding the *cis*-acting function for SL I and SL II in the context of subgenome and genome, these results may suggest that the individual SL is required for the template switching that is essential for the synthesis of (−)-strand sgmRNA from genome but the individual SL is not critical for the subsequent amplification of sgmRNA (−) strand using sgmRNA as a template.

Based on the secondary structure model of MHV 3' UTR proposed by Züst* et al.* [45], two helical stems formed at the terminus of MHV 3' UTR are functionally important for the initiation of (−)-strand RNA synthesis. Subsequent analysis of the two stems by Liu* et al.* [54] with reverse genetic approaches suggests that the first stem (designated S3) in which nts 0 to −9 (0 indicates the 5'-most nt of the poly(A) tail and −9 indicates the ninth nt counted from poly(A) tail) are base-paired with loop 1 of the pseudoknot stem is required for virus viability; however, disruptions in the second stem (designated S4) in which nts −18 to −29 are base-paired with nts downstream of the pseudoknot stem 2 generate both viable and lethal mutants. Interestingly, in the present study the deletion mutant s3'Δ15, in which the 3'-most 15 nts were deleted, still maintained the ability to synthesize its (−)-strand counterpart and therefore does not seem to support the Züst model. However, folding of this deleted mutant by Mfold algorithm revealed that the partial S3 and entire S4 are still maintained although the base-paired sequence in S4 is altered. Since the secondary structures of S3 and S4 are not impaired dramatically, this may explain why the mutant s3'Δ15 still supports the (−)-strand sgmRNA synthesis. In contrast, folding of the mutants s3'Δ30–15 and pseudoknot-deleted sΔP by Mfold algorithm showed that the structures of both S3 and S4 are disrupted and this may account for the significant inhibition of (−)-strand sgmRNA synthesis for these mutants. Therefore, regarding the secondary structures of the two helical stems S3 and S4 in the (−)-strand sgmRNA synthesis, the results in the present study are consistent with the conclusions by Liu* et al.* [54] and support the model proposed by Züst* et al.* [45].

It has been demonstrated that the 3'-most nucleotide species in BCoV DI RNA affects the efficiency of (−)-strand RNA synthesis [32]. The same results were obtained for sgmRNA 7 in the present study (Figure 5). However, (−)-strand RNA synthesis from mutant s3'Δ15 was maintained even though the 3'-most 15 nucleotides had been deleted. Upon examination it was learned the 3'-most nucleotide in mutant s3'Δ15 (cytosine) was the same as that on wt sBM25A. Since this nucleotide is the same, and since the secondary structures of S3 and S4 are not seriously impaired as discussed above, these features may explain why this mutant still maintained the ability to synthesize its (−)-strand counterpart.

The terminal structures, including the 5'-terminal leader sequence and 3'-terminal 55 nts, on sgmRNA apparently play critical roles with regard to (−)-strand sgmRNA synthesis. It is logical that the initiation of (−)-strand sgmRNA synthesis is triggered from the 3' terminus of sgmRNA and that mutations in the 3'-terminal region may be detrimental to the synthesis of (−)-strand sgmRNA. However, the question remains as to how the sgmRNA 5'-terminal structures affect (−)-strand sgmRNA synthesis, as deletion of the 5' leader sequence impaired the synthesis of (−)-strand sgmRNA in this study. Since the interactions between the 5' and 3' termini of the virus genome have been suggested to be required for the initiation of replication in RNA viruses [2,9,12,46,55,56,57,58,59,60,61], including coronaviruses [2,9,12,46,57], and both the 5'-terminal leader sequence and 3'-terminal 55-nt sequence in the context of (+)-strand sgmRNA are required for the (−)-strand sgmRNA synthesis, we speculate that the interactions between the 5' and 3' termini of (+)-strand sgmRNA may explain why the 5'-terminal leader sequence is critical for the synthesis of (−)-strand sgmRNA. With this interpretation, we would like to note that since other 5'-terminal structures between BCoV DI RNA and sgmRNA differ (Figure 1A), the *cis*-acting requirements within the 5' and 3' termini and the nature of the assembled replication complexes leading to the initiation of (−)-strand RNA synthesis probably also vary between the two RNA species. Therefore, this variation in structure may explain why nts −55 to −35 and the last 15 nts within the 3'-terminal 55-nt sequence are required for efficient (−)-strand BCoV DI RNA synthesis [32], but are dispensable for (−)-strand sgmRNA synthesis in the present study (Figure 4).

In this study, we systematically examined the *cis*-acting elements on sgmRNA and found that the 5'- and 3'-terminal sequences on sgmRNA 7 harbor *cis*-acting elements essential for efficient (−)-strand sgmRNA synthesis in BCoV. These findings have extended our knowledge of coronavirus replication. Furthermore, the reasons for the various observations discussed above regarding the requirements of 5'- and 3'-terminal *cis*-acting elements for the synthesis of (−)-strand RNA from genomic RNA and sgmRNA remain unclear; however, we speculate that the different experimental approaches may be largely responsible for the different outcomes. Therefore, further studies with similar systems are required in order to explain the various results.

## Supplementary Files

Supplementary File 1

## References

[1] Brian D.A., Baric R.S. (2005). Coronavirus genome structure and replication. Curr. Top. Microbiol. Immunol..

[2] Enjuanes L., Almazan F., Sola I., Zuniga S. (2006). Biochemical aspects of coronavirus replication and virus-host interaction. Annu. Rev. Microbiol..

[3] Pasternak A.O., Spaan W.J., Snijder E.J. (2006). Nidovirus transcription: How to make sense...?. J. Gen. Virol..

[4] Van Vliet A.L., Smits S.L., Rottier P.J., de Groot R.J. (2002). Discontinuous and non-discontinuous subgenomic rna transcription in a nidovirus. EMBO J..

[5] Ozdarendeli A., Ku S., Rochat S., Williams G.D., Senanayake S.D., Brian D.A. (2001). Downstream sequences influence the choice between a naturally occurring noncanonical and closely positioned upstream canonical heptameric fusion motif during bovine coronavirus subgenomic mrna synthesis. J. Virol..

[6] Sawicki S.G., Sawicki D.L. (1990). Coronavirus transcription: Subgenomic mouse hepatitis virus replicative intermediates function in rna synthesis. J. Virol..

[7] Sawicki S.G., Sawicki D.L. (2005). Coronavirus transcription: A perspective. Curr. Top. Microbiol. Immunol..

[8] Sethna P.B., Hung S.L., Brian D.A. (1989). Coronavirus subgenomic minus-strand rnas and the potential for mrna replicons. Proc. Natl. Acad. Sci. USA.

[9] Sola I., Moreno J.L., Zuniga S., Alonso S., Enjuanes L. (2005). Role of nucleotides immediately flanking the transcription-regulating sequence core in coronavirus subgenomic mrna synthesis. J. Virol..

[10] Wu H.Y., Brian D.A. (2007). 5'-proximal hot spot for an inducible positive-to-negative-strand template switch by coronavirus RNA-dependent RNA polymerase. J. Virol..

[11] Wu H.Y., Ozdarendeli A., Brian D.A. (2006). Bovine coronavirus 5'-proximal genomic acceptor hotspot for discontinuous transcription is 65 nucleotides wide. J. Virol..

[12] Zuniga S., Sola I., Alonso S., Enjuanes L. (2004). Sequence motifs involved in the regulation of discontinuous coronavirus subgenomic RNA synthesis. J. Virol..

[13] Chang R.Y., Hofmann M.A., Sethna P.B., Brian D.A. (1994). A cis-acting function for the coronavirus leader in defective interfering RNA replication. J. Virol..

[14] Wu H.Y., Brian D.A. (2010). Subgenomic messenger RNA amplification in coronaviruses. Proc. Natl. Acad. Sci. USA.

[15] Baric R.S., Fu K., Schaad M.C., Stohlman S.A. (1990). Establishing a genetic recombination map for murine coronavirus strain a59 complementation groups. Virology.

[16] Fu K., Baric R.S. (1994). Map locations of mouse hepatitis virus temperature-sensitive mutants: Confirmation of variable rates of recombination. J. Virol..

[17] Koetzner C.A., Parker M.M., Ricard C.S., Sturman L.S., Masters P.S. (1992). Repair and mutagenesis of the genome of a deletion mutant of the coronavirus mouse hepatitis virus by targeted RNA recombination. J. Virol..

[18] Masters P.S., Koetzner C.A., Kerr C.A., Heo Y. (1994). Optimization of targeted RNA recombination and mapping of a novel nucleocapsid gene mutation in the coronavirus mouse hepatitis virus. J. Virol..

[19] Fu K., Baric R.S. (1992). Evidence for variable rates of recombination in the mhv genome. Virology.

[20] Garcia-Arriaza J., Ojosnegros S., Davila M., Domingo E., Escarmis C. (2006). Dynamics of mutation and recombination in a replicating population of complementing, defective viral genomes. J. Mol. Biol..

[21] Eckerle L.D., Lu X., Sperry S.M., Choi L., Denison M.R. (2007). High fidelity of murine hepatitis virus replication is decreased in nsp14 exoribonuclease mutants. J. Virol..

[22] Brown C.G., Nixon K.S., Senanayake S.D., Brian D.A. (2007). An RNA stem-loop within the bovine coronavirus nsp1 coding region is a cis-acting element in defective interfering RNA replication. J. Virol..

[23] Chang R.Y., Krishnan R., Brian D.A. (1996). The ucuaaac promoter motif is not required for high-frequency leader recombination in bovine coronavirus defective interfering RNA. J. Virol..

[24] Kang H., Feng M., Schroeder M.E., Giedroc D.P., Leibowitz J.L. (2006). Putative *cis*-acting stem-loops in the 5' untranslated region of the severe acute respiratory syndrome coronavirus can substitute for their mouse hepatitis virus counterparts. J. Virol..

[25] Lin Y.J., Lai M.M. (1993). Deletion mapping of a mouse hepatitis virus defective interfering RNA reveals the requirement of an internal and discontiguous sequence for replication. J. Virol..

[26] Lin Y.J., Liao C.L., Lai M.M. (1994). Identification of the *cis*-acting signal for minus-strand RNA synthesis of a murine coronavirus: Implications for the role of minus-strand RNA in RNA replication and transcription. J. Virol..

[27] Raman S., Bouma P., Williams G.D., Brian D.A. (2003). Stem-loop iii in the 5' untranslated region is a *cis*-acting element in bovine coronavirus defective interfering RNA replication. J. Virol..

[28] Raman S., Brian D.A. (2005). Stem-loop iv in the 5' untranslated region is a *cis*-acting element in bovine coronavirus defective interfering RNA replication. J. Virol..

[29] Spagnolo J.F., Hogue B.G. (2000). Host protein interactions with the 3' end of bovine coronavirus RNA and the requirement of the poly(a) tail for coronavirus defective genome replication. J. Virol..

[30] Van der Most R.G., Luytjes W., Rutjes S., Spaan W.J. (1995). Translation but not the encoded sequence is essential for the efficient propagation of the defective interfering rnas of the coronavirus mouse hepatitis virus. J. Virol..

[31] Williams G.D., Chang R.Y., Brian D.A. (1999). A phylogenetically conserved hairpin-type 3' untranslated region pseudoknot functions in coronavirus RNA replication. J. Virol..

[32] Liao W.Y., Ke T.Y., Wu H.Y. (2014). The 3'-terminal 55 nucleotides of bovine coronavirus defective interfering RNA harbor *cis*-acting elements required for both negative- and positive-strand RNA synthesis. PLoS One.

[33] Gustin K.M., Guan B.J., Dziduszko A., Brian D.A. (2009). Bovine coronavirus nonstructural protein 1 (p28) is an RNA binding protein that binds terminal genomic *cis*-replication elements. J. Virol..

[34] Hsue B., Hartshorne T., Masters P.S. (2000). Characterization of an essential RNA secondary structure in the 3' untranslated region of the murine coronavirus genome. J. Virol..

[35] Hsue B., Masters P.S. (1997). A bulged stem-loop structure in the 3' untranslated region of the genome of the coronavirus mouse hepatitis virus is essential for replication. J. Virol..

[36] Goebel S.J., Miller T.B., Bennett C.J., Bernard K.A., Masters P.S. (2007). A hypervariable region within the 3' *cis*-acting element of the murine coronavirus genome is nonessential for RNA synthesis but affects pathogenesis. J. Virol..

[37] Lapps W., Hogue B.G., Brian D.A. (1987). Sequence analysis of the bovine coronavirus nucleocapsid and matrix protein genes. Virology.

[38] King B., Brian D.A. (1982). Bovine coronavirus structural proteins. J. Virol..

[39] Wu H.Y., Ke T.Y., Liao W.Y., Chang N.Y. (2013). Regulation of coronaviral poly(a) tail length during infection. PLoS One.

[40] (1996). GraphPad Prism.

[41] Hofmann M.A., Sethna P.B., Brian D.A. (1990). Bovine coronavirus mrna replication continues throughout persistent infection in cell culture. J. Virol..

[42] Schenborn E.T., Mierendorf R.C. (1985). A novel transcription property of sp6 and t7 RNA polymerases: Dependence on template structure. Nucleic Acids Res..

[43] Lai M.M. (1992). Genetic recombination in RNA viruses. Curr. Top. Microbiol. Immunol..

[44] Ke T.Y., Liao W.Y., Wu H.Y. (2013). A leaderless genome identified during persistent bovine coronavirus infection is associated with attenuation of gene expression. PLoS One.

[45] Zust R., Miller T.B., Goebel S.J., Thiel V., Masters P.S. (2008). Genetic interactions between an essential 3' *cis*-acting RNA pseudoknot, replicase gene products, and the extreme 3' end of the mouse coronavirus genome. J. Virol..

[46] Li L., Kang H., Liu P., Makkinje N., Williamson S.T., Leibowitz J.L., Giedroc D.P. (2008). Structural lability in stem-loop 1 drives a 5' utr-3' utr interaction in coronavirus replication. J. Mol. Biol..

[47] Liu P., Li L., Keane S.C., Yang D., Leibowitz J.L., Giedroc D.P. (2009). Mouse hepatitis virus stem-loop 2 adopts a uynmg(u)a-like tetraloop structure that is highly functionally tolerant of base substitutions. J. Virol..

[48] Liu P., Li L., Millership J.J., Kang H., Leibowitz J.L., Giedroc D.P. (2007). A u-turn motif-containing stem-loop in the coronavirus 5' untranslated region plays a functional role in replication. RNA.

[49] Liu Q., Johnson R.F., Leibowitz J.L. (2001). Secondary structural elements within the 3' untranslated region of mouse hepatitis virus strain jhm genomic RNA. J. Virol..

[50] Guan B.J., Su Y.P., Wu H.Y., Brian D.A. (2012). Genetic evidence of a long-range RNA-RNA interaction between the genomic 5' untranslated region and the nonstructural protein 1 coding region in murine and bovine coronaviruses. J. Virol..

[51] Guan B.J., Wu H.Y., Brian D.A. (2011). An optimal *cis*-replication stem-loop iv in the 5' untranslated region of the mouse coronavirus genome extends 1. J. Virol..

[52] Chang R.Y., Brian D.A. (1996). *Cis* requirement for *n*-specific protein sequence in bovine coronavirus defective interfering RNA replication. J. Virol..

[53] Liao C.L., Lai M.M. (1995). A *cis*-acting viral protein is not required for the replication of a coronavirus defective-interfering RNA. Virology.

[54] Liu P., Yang D., Carter K., Masud F., Leibowitz J.L. (2013). Functional analysis of the stem loop s3 and s4 structures in the coronavirus 3'utr. Virology.

[55] Alvarez D.E., Filomatori C.V., Gamarnik A.V. (2008). Functional analysis of dengue virus cyclization sequences located at the 5' and 3'utrs. Virology.

[56] Alvarez D.E., Lodeiro M.F., Luduena S.J., Pietrasanta L.I., Gamarnik A.V. (2005). Long-range RNA-RNA interactions circularize the dengue virus genome. J. Virol..

[57] Enjuanes L., Almazan F., Sola I., Zuniga S., Alvarez E., Reguera J., Capiscol C. (2006). Biochemical aspects of coronavirus replication. Adv. Exp. Med. Biol..

[58] Filomatori C.V., Lodeiro M.F., Alvarez D.E., Samsa M.M., Pietrasanta L., Gamarnik A.V. (2006). A 5' RNA element promotes dengue virus RNA synthesis on a circular genome. Genes Dev..

[59] Herold J., Andino R. (2001). Poliovirus RNA replication requires genome circularization through a protein-protein bridge. Mol. Cell.

[60] Ooms M., Abbink T.E., Pham C., Berkhout B. (2007). Circularization of the HIV-1 RNA genome. Nucleic Acids Res..

[61] Villordo S.M., Gamarnik A.V. (2009). Genome cyclization as strategy for flavivirus RNA replication. Virus Res..

