# Supplementary material for: Identification of Cis-Acting Elements on Positive-Strand Subgenomic mRNA Required for the Synthesis of Negative-Strand Counterpart in Bovine Coronavirus
Source: Viruses. 2014 Jul 30;6(8):2938–59. doi: 10.3390/v6082938 (PMC4147681; doi:10.3390/v6082938)

Supplementary Materials

Identification of *Cis*-Acting Elements on Positive-Strand Subgenomic mRNA Required for the Synthesis of Negative-Strand Counterpart in Bovine Coronavirus

Po-Yuan Yeh and Hung-Yi Wu

Table S1. Oligonucleotides used in this study

Oligonucleotide[a]	Polarity[b]	Sequence (5' → 3')	Position
pGEMNDEI(–)[c]	+	GAGAGTGCACCATATG	
△NL (+)	–	GACATCCTTAAAGTTTAGACCCTATAGTGAGTCGTATTAC	
△SLI (+)	–	TAAAAAGATCTAACAAGAGACTATAGTGAGAGTCGTATTACA	
△SLII (+)	–	CCTTAAAGTTTAGATCAGTGAAGCGGGATG	
sΔB(+)	–	CAAGACATCCATTCTGAATATTTCTGAGGTGTC	
s△P (+)	–	CTAATTGATACAGGGTTGTCCTATCCCGACTTTC	
s3'△15 (+)	–	ACGCGTTTTTTTTTTTTTTTTTTTTTTTTTGGCCATGATCAACTTC	
s3'Δ55 (+)	–	ACGCGTTTTTTTTTTTTTTTTTTTTTTTTTAATAGTACCCTGATGTG	
s3'△55-45 (+)	–	CATTCATTTACTAGGAATAGTACCCTGATGTGAGC	
s3'△55-40 (+)	–	CTTCATTCATTTAAATAGTACCCTGATGTGAGC	
s3'△55-30 (+)	–	GGCCATGATCAACTTAATAGTACCCTGATGTGAGC	
s3'Δ30-15 (+)	–	GTGATTCTTCCAATTCATTCATTTACTAGGGC	
sgmA' (+)	–	ACGCGTTTTTTTTTTTTTTTTTTTTTTTTTTTGATTCTTCCAATTGGCC	
sgmU' (+)	–	ACGCGTTTTTTTTTTTTTTTTTTTTTTTTTATGATTCTTCCAATTGGCC	
sgmG' (+)	–	ACGCGTTTTTTTTTTTTTTTTTTTTTTTTTCTGATTCTTCCAATTGGCC	
leader20(–)	+	GATTGTGAGCGATTTGCGTG	1-20[d]
M(+)	–	GTTCCATTCCTTTAGGAATTTAATAG	28746-28771[d]
M3 (–)	+	GGGTTCTGGCATGGACACCGC	29345-29365[d]
MHV3'UTR 3 (–)	+	GTCCTACGTCTAACCATAAGAACG	31213-31236[e]
MHV3'UTR 6 (–)	+	CCTGGGAAGAGCTCACATCAGG	31250-31271[e]
BCV 23-40 (+)	–	CCCGCTTCACTGATCTCT	23-40[d]
BCV 29-54 (+)	–	GATCTAACAAGAGTCAGTGAAGCG	29-54[d]
TGEV 7 (-)	+	TCTGGGTTGCCAAGGATGGTGCCATG	1098-1123[f]
TGEV 8 (+)	–	CATGGCACCATCCTTGGCAACCCAGA	1098-1123[f]
DI reverse (+)[c]	–	CACAGGAAACAGCTATGACC	
BCVN (+)	–	CCAGAACGATTTCCAAAGGACGCTCT	29430-29455[d]
18SrRNA (+)	–	GCCTGCTGCCTTCCTTGGATCTGGTAGCC	552-580[g]
18SrRNA (–)	+	CCCATTCGAACGTCTGCCCTATC	440-463[g]
MHV3'UTR2 (+)	–	CCTATCGCCGTTCTTATGGTTAGACG	31219-31244[e]
Taqman probe-5 (–)	+	TGAGCGATTTGCGTGCG	6-22[d]
Taqman probe-3 (–)	+	TAACCATAAGAACGGCGATAGGCGC	31223-31247[e]
MHV3'UTR-DR(–)	+	GATTGCAAAATAGAGAATGTG	31175-31197[e]
MHV3'UTR-DR(+)	–	AATAGTACCCTGATGTGA	31262-31279[e]

[a] The positive and negative symbols in the oligonucleotide names indicate the polarities of the nucleic acids to which the oligonucleotides anneal. Oligonucleotides named with a negative symbol used for mutagenesis in the text have a sequence complementaery to a positive-sense oligonucelotide of the same name. [b] Polarity of the oligonucleotide relative to the positive-strand viral genome. [c] Indicates the oligonucleotide which anneals to pGEM3Zf(-) vector (Promega). [d] BCoV-Mebus: GenBank accession number U00735.2; [e] MHV-A59: GenBank accession number NC_001846.1; [f] Indicates the reporter sequence in BCoV DI RNA which anneals to oligonucleotide; [g] 18S rRNA: GenBank accession number M10098.1.

Figure S1. Comparison of the efficiency of the (–)-strand RNA synthesis between sgmRNA 7 and BCoV DI RNA without the strategy of head-to-tail ligation. (**A**) RT-PCR product was observed from BCoV-infected and BM25A- or sBM25A-transfected cells (lanes 2 and 3) but not from control groups: lane 4, total cellular RNA from mock-infected cells; lane 5, total cellular RNA from BCoV-infected and mock-transfected cells. RT-PCR products of ~100 bp were also observed from mock-infected and BM25A- or sBM25A-transfected cells (lanes 6 and 7). (**B**) The relative efficiency of (–)-strand RNA synthesis from constructs BM25A and sBM25A, as measured by RT-qPCR without the step of head-to-tail ligation. Control A: total cellular RNA from mock-infected cells. Control B: total cellular RNA from BCoV-infected cells. Control C: total cellular RNA from BM25A-transfected mock-infected cells. Control D: total cellular RNA from sBM25A -transfected mock-infected cells. M (lanes 1 and 8), ds DNA size markers in nt pairs. The values (**B**) represent the mean±SD of three individual experiments. * $p < 0.05$.

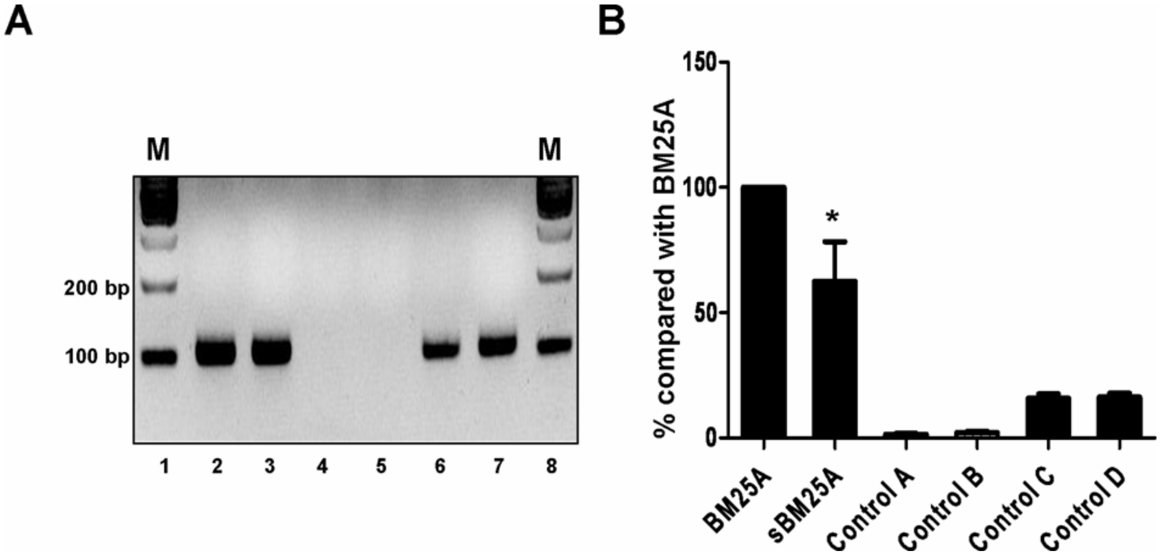

Figure S2. Analysis of the positive-strand RNA synthesis at 48 hpi of VP1 by Northern blot assay with 18S rRNA and M sgmRNA as internal controls.

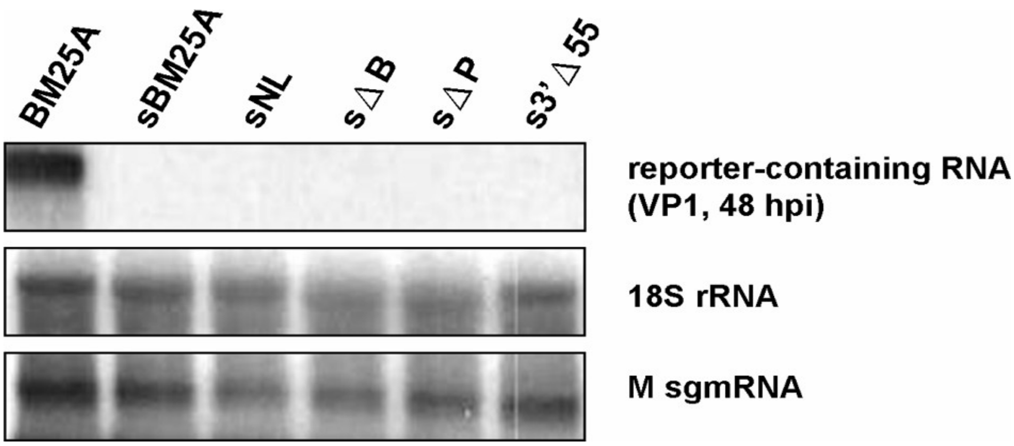

Supplement: Supplementary File 1 [file viruses-06-02938-s001.pdf]
